# Grafting the ALFA tag for structural studies of aquaporin Z

**DOI:** 10.1016/j.yjsbx.2024.100097

**Published:** 2024-02-02

**Authors:** Lauren Stover, Hanieh Bahramimoghaddam, Lie Wang, Samantha Schrecke, Gaya P. Yadav, Ming Zhou, Arthur Laganowsky

**Affiliations:** a Department of Chemistry, Texas A&M University, College Station, TX 77843, United States; b Verna and Marrs McLean Department of Biochemistry and Molecular Biology, Baylor College of Medicine, Houston, TX 77030, United States; c Laboratory for Biomolecular Structure and Dynamics (LBSD), Department of Biochemistry and Biophysics, Texas A&M University, College Station, TX 77843, United States

## Abstract

Aquaporin Z (AqpZ), a bacterial water channel, forms a tetrameric complex and, like many other membrane proteins, activity is regulated by lipids. Various methods have been developed to facilitate structure determination of membrane proteins, such as the use of antibodies. Here, we graft onto AqpZ the ALFA tag (AqpZ-ALFA), an alpha helical epitope, to make use of the high-affinity anti-ALFA nanobody (nB). Native mass spectrometry reveals the AqpZ-ALFA fusion forms a stable, 1:1 complex with nB. Single-particle cryogenic electron microscopy studies reveal the octameric (AqpZ-ALFA)_4_(nB)_4_ complex forms a dimeric assembly and the structure was determined to 1.9 Å resolution. Dimerization of the octamer is mediated through stacking of the symmetrically bound nBs. Tube-like density is also observed, revealing a potential cardiolipin binding site. Grafting of the ALFA tag, or other epitope, along with binding and association of nBs to promote larger complexes will have applications in structural studies and protein engineering.

## Highlights

• Nanobodies and antibodies are often used to improve crystal packing, trap a particular conformation, and/or increase particle size for structural studies of membrane proteins.
• Application of the ALFA tag, an alpha helical epitope, and its high-affinity nanobody binder for structural studies of the bacterial water channel AqpZ.
• The anti-ALFA nanobody promotes oligomerization, forming a large 16-mer complex that is ideal for cryo-electron microscopy studies.
• The large complex led to a 1.9 Å resolution structure of AqpZ and revealed a cardiolipin binding site.
• Grafting the ALFA tag onto membrane proteins offers another approach to the growing structural biology toolkit.

## Graphical abstract

**Figure:**
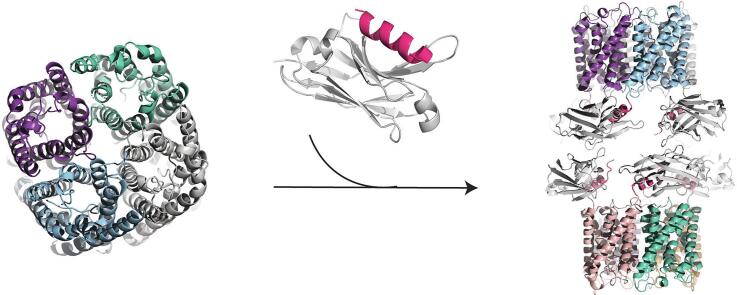

## Introduction

Aquaporins are responsible for the selective, passive transport of water and small amphiphiles across the biological membrane. (Borgnia et al., 1999) The two subfamilies of the aquaporin family are the aquaporins that are selective for water and aquaglyceroporins that transport water and small molecules, such as glycerol. The bacterial aquaporin AqpZ forms a tetrameric complex that efficiently transports water. (Calamita et al., 1995, Borgnia and Agre, 2001) The monomeric subunit adopts an aquaporin fold composed of six transmembrane helices and two-half spanning helices (TM1-TM8). (Savage et al., 2003, Jiang et al., 2006) The structure adopts a pseudo-two-fold symmetry with the water channel containing two highly conserved regions. Located at the narrowest point, and centrally between the extracellular vestibule and NPA (asp-pro-ala) motif, is the selectivity filter formed by sidechains of Phe43, His174, and Arg189. Arg189 is thought to regulate water transport by adopting different conformations that promote or disrupt water flow. (Jiang et al., 2006, Wang et al., 2005) The other conserved region is the NPA motif, which are found in the amino-terminal ends of M3 and M7 half helices, that interact in the center of the water channel. The asparagine residues within the NPA motif are responsible for polarizing the orientation of water molecules. (Tajkhorshid et al., 2002) Despite advances in determining structures of AqpZ and those of other aquaporins, it remains poorly understood how lipids regulate water transport.

Several approaches have been employed to improve crystal packing, trap a particular conformation, and/or increase particle size for structural studies of membrane proteins. (Wentinck et al., 2022, Bukowska and Grutter, 2013) One approach involves raising antibodies to protein targets. A classic example is the complex of the potassium channel KcsA in complex with a fragment antibody, which led to a high-resolution structure revealing ion coordination and hydration. (Zhou et al., 2001) Camelid single-domain antibody fragments, often referred to as nanobodies, (Hamers-Casterman et al., 1993) have also been employed to facilitate structure determination, such as stabilizing the active state of a G-protein coupled receptor (GPCR). (Rasmussen et al., 2011) Synthetic nanobody libraries and methods have been developed to aid the identification of conformationally selective nanobodies. (McMahon et al., 2018, Zimmermann et al., 2020) Other strategies involve the fusion of soluble proteins within loops of membrane proteins, (Prive et al., 1994) which has been an effective strategy for structure determination of GPCRs. (Rosenbaum et al., 2007) Although the choice of loop fusion proteins range in size and structure, they share a relative short separation of N- and C-termini. (Chun et al., 2012) Synthetic antibodies have been developed that bind BRIL, an engineered variant of apocytochrome b562a that is commonly used as a loop fusion protein, to increase particle size for single-particle cryogenic electron microscopy (cryoEM) studies. (Mukherjee et al., 2020) Along these lines, there has been a recent effort to develop methods to increase the size of nanobodies bound to protein targets by decorating with other molecules or grafting onto selected protein scaffolds. (Bloch et al., 2021, Wu and Rapoport, 2021, Uchanski et al., 2021) Another strategy involves grafting alpha-helical epitopes onto alpha-helices of proteins providing a docking site for antibody binding. (Kim et al., 2019, Stockbridge and Wolfenden, 2009).

## Results

To facilitate structure determination and orientation of particles, we grafted the ALFA tag, a short alpha helical peptide with a minimal sequence of SRLEEELRRRLTE, (Petukhov et al., 2009, Gotzke et al., 2019) onto the cytoplasmic-exposed, C-terminus of AqpZ (AqpZ-ALFA). The fusion was initially modeled as a continuation of the C-terminal transmembrane helix to aid registration and position of the graft (Fig. S1). The ALFA epitope is known to bind an anti-ALFA nanobody (nB) with a reported dissociation constant of ∼ 26 pM, (Gotzke et al., 2019) which we would make use of as a fiducial marker of the cytoplasmic-facing side of AqpZ. Similar fusion strategies have been employed to enable structural studies. (Kim et al., 2019, McIlwain et al., 2021) Samples of AqpZ-ALFA readily associated with the nB, forming a uniform complex in the C8E4 detergent (Fig. S2). Interestingly, we noticed the AqpZ-ALFA-nB was temperature sensitive; at cold temperatures the complex would aggregate forming a white cloudy solution.

**Fig. 1:**
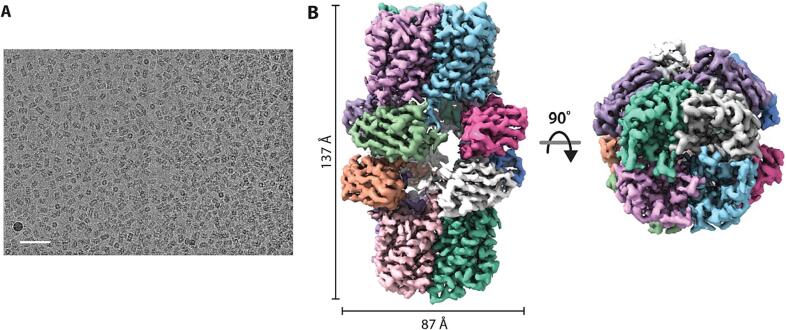
Structure of the AqpZ-ALFA-nB complex. (A) Micrograph of AqpZ-ALFA-St with nanobody. A 50 nm scale bar is shown. (B) CryoEM reconstruction (1.9 Å) map with subunits shown in different colors. Two views of the complex are shown along with dimensions of the complex.

We then prepared the AqpZ-ALFA-nB complex for cryoEM in the presence of 5-fold molar excess of cardiolipin, a lipid that has been identified to regulate water transport activity. (Laganowsky et al., 2014) The complex was prepared at room temperature and applied to a grid incubated at 5 °C prior to blotting and flash freezing. Micrographs showed the AqpZ-ALFA-nB complex assembled into a larger, dimeric configuration (Fig. 1A). The structure of the dimeric AqpZ-ALFA-nB complex was determined to a resolution of 1.9 Å with D4 symmetry (Fig. 1B). The structure of AqpZ is like those previously reported. (Savage et al., 2003).

**Fig. 2:**
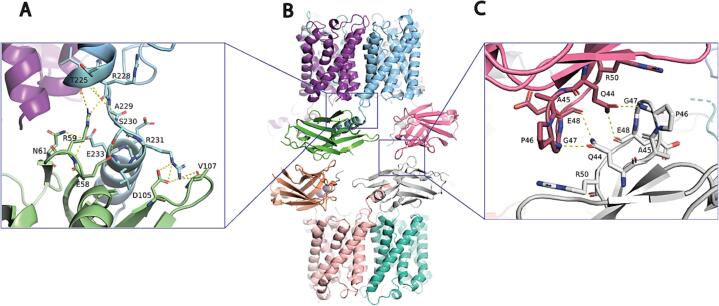
Structure of the AqpZ-ALFA-nB complex. (A) The molecular interactions between AqpZ (cyan) with the ALFA peptide bound to nB (light green). Bonds are shown as dashed yellow lines and residues are labeled. (B) Cartoon view of the AqpZ-nB complex. (C) The nB-nB interface responsible for dimerization of the AqpZ-ALFA-nB complex. Shown as described in panel A.

There are several structural features that contribute to the dimerization of the Aqpz-nB complex. First, the structure of the ALFA-nB component is reminiscent of the structure with the ALFA peptide, in which the N-terminus of the ALFA peptide forms a loop stabilized by interactions with the nB (Fig. 2A). The ALFA-nB structure folds back onto AqpZ and is stabilized by Asn61 of the nB, forming a hydrogen bond with the backbone amide of Arg3 in the N-terminal helix of AqpZ. An additional hydrogen bond is formed from Lys79 of AqpZ with the backbone carbonyl of Leu240 in the nB. The dimeric assembly of the AqpZ-ALFA-nB complex is mediated through nB-nB interactions (Fig. 2C). More specifically, the surface area buried between nB-nB contacts is ∼ 259 Å^2^, and Gln44 within one nB forms hydrogen bonds with the backbone amide of Gly47 and carbonyl of Glu48 of the interacting nB. Moreover, the nB-nB interactions observed here are not similar to the crystal packing contacts observed in the crystal structure of nB-ALFA complex. (Gotzke et al., 2019) These results demonstrate the utility of the ALFA-nB in facilitating structure determination.

**Fig. 3:**
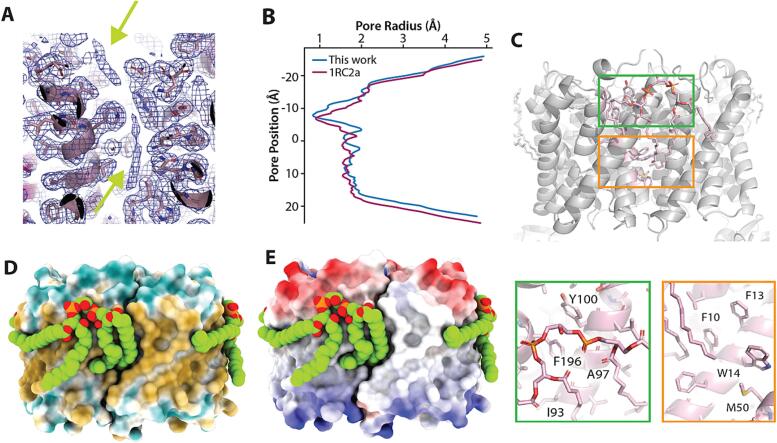
Insight into water transport and CDL interactions with AqpZ. A) Density (contoured at 4 sigma) of water lining the water channel within an AqpZ protomer. B) Computed pore diameter of the water channel using HOLE2.^28^ The channel diameter for chain A of PDB 1RC2 is also shown. C) Molecular views of CDL bound to AqpZ. Shown below are two views highlighting headgroup and acyl chain interactions with AqpZ. Residue labels are shown. D) Hydrophilic and hydrophobic surfaces of AqpZ colored blue and gold, respectively. E) Coulombic electrostatic potential (scale bar −10 to + 10 as computed by ChimeraX^29^) for negative and positive charges colored red and blue, respectively.

Additional density is also observed in the structure of the AqpZ-ALFA-nB complex (Fig. 3). Discontinuous density centered within each AqpZ protomer lines the water transport channel (Fig. 3A). This density is comparable to those observed for other AqpZ structures (Fig. S4). Analysis of the water transport channel using HOLE (Smart et al., 1996) reveals a constriction around Phe43, the location of the selectivity filter, and this is the same region where the discontinuity in density is observed (Fig. 3B). The channel adopts a structure like chain B of PDB 1RC2 (Savage et al., 2003) that also differs from chain A of the same structure. Lipid-like density is observed near the interface of protomers, located on the periplasmic-facing leaflet of the inner membrane (Fig. S5). A CDL molecule could be modeled into parts of this density, positioning the lipid onto the lipid-facing protomer surface (Fig. 3C-E). This results in one CDL molecule bound per AqpZ subunit. Part of the CDL acyl chains nestle into a hydrophobic pocket, one of which is formed by Phe10, Phe13, and Trp14 (Fig. 3C, lower panel). The acyl chains of the modeled CDL cradle a hydrophobic and neutral surface of AqpZ (Fig. 3D-E). The headgroup of CDL is positioned at the hydrophilic and hydrophobic interface (Fig. 3D-E).

## Discussion

The AqpZ-ALFA-nB structure reveals a CDL binding site that may regulate water transport. Here, the ALFA-tag is used as a fiducial marker for cryoEM studies and the nB also facilitated the assembly of a larger complex with D4 symmetry. Density lines the water channel of each AqpZ protomer, and the largest pore constriction occurs at the selectivity filter. This symmetric arrangement is similar to a previous structural study, (Savage et al., 2003) but differs from another where the tetrameric AqpZ assembly adopts different structures. (Jiang et al., 2006) The CDL binding site lies within the periplasmic-facing leaflet of the inner membrane. Interestingly, some of the acyl chains of CDL penetrate an opening located at a subunit-subunit interface. This opening is lined with hydrophobic residues, such as Trp14 that when mutated alters CDL binding. (Jayasekera et al., 2023) It is also important to note that the samples of AqpZ have been optimized using native MS and do not contain endogenous lipids. Thus, the density observed is consistent with TOCDL that was added back to the sample. Nevertheless, a question that remains is how does CDL enhance water activity? It is possible CDL promotes the water channel to adopt water conducting channel, as we observe R189 populating an upward, conductive conformation (Jiang et al., 2006).

The ALFA tag along with the high affinity nB has potential to be adapted to other protein complexes. The structure of the ALFA peptide bound to nB shows the alpha helical epitope is flanked by residues that are unstructured. (Gotzke et al., 2019) The discontinuity of the alpha helix is promoted by several interactions with the nB. The grafted ALFA tag on AqpZ adopts the same structure for ALFA-nB complex but several key interactions are formed between the docked nB and AqpZ. This stabilizes the nB which in turn facilitates the temperature-driven association of the symmetrically bound nBs, promoting a large, dimeric complex that is ideal for cryoEM studies. One can envision using the transmembrane-ALFA peptide bound to nB structure to find suitable grafting conditions. However, the discontinuity of the junction between the transmembrane helix and ALFA tag will have to be taken into consideration. It is likely that different grafts of the ALFA tag will have to be explored, as demonstrated for the MPER peptide. (McIlwain et al., 2021) Approaches to introduce symmetry through the introduction of disulfide bonds and metal binding sites has proved useful for structural studies. (Banatao et al., 2006, Laganowsky et al., 2011, Salgado et al., 2010, Salgado et al., 2010) The structure presented here shows that nB is prone to self-associating, leading to the formation of a larger, dimeric complex, an ideal situation for structural studies. There is also opportunity for engineering nBs to associate and form distinct complexes. (Lai et al., 2012, Yeates, 2017) In closing, the ALFA tag offers another approach to the structural biology toolkit.

## Materials and methods

*AqpZ-ALFA and anti-ALFA expression plasmids.* The pET-based expression plasmid (Laganowsky et al., 2014) to express *Ecoli* AqpZ (Uniprot P60844) with a TEV protease C-terminal fusion to superfolder GFP (Pedelacq et al., 2006) and 6x His-tag was modified for cryoEM studies. The C-terminal fusion was replaced with an ALFA tag followed by a flexible linker and Strep-tag II affinity tag. The resulting fusion sequence is IYRTLLRA**SRLEEELRRRLTE**PGGGPGaswshpqfek, where the underlined sequence denotes C-terminal residues of AqpZ, bold sequence ALFA tag, and the lowercase sequence the Strep-tag II. The fusion sequence was introduced using primers designed with the NEB BaseChanger website (New England Biolabs) and the KLD enzyme mix (New England Biolabs) according to the manufacturer’s protocol. The anti-ALFA nanobody sequence with an N-terminal 10x His-tag and bdSUMO fusion was synthesized and cloned into pET29b (Twist Biosciences). The AqpZ-ALFA and anti-ALFA nanobody expression plasmids have been deposited at AddGene with accession numbers 212,310 and 212311, respectively.

*AqpZ-ALFA expression and purification.* The AqpZ-ALFA expression plasmid was transformed into BL21-A1 cells (Invitrogen). A single colony was used to inoculate 50 mL of LB media and grown overnight at 37 °C while shaking. The overnight culture was used to inoculate TB media (7 mL per 1L of TB in a 2L Erlenmeyer flask). Once the OD_600_ reached between 0.6 and 0.8, arabinose was added to a final concentration of 0.2 % to induce protein expression and the temperature was lowered to 20 °C and shaken overnight. The following day the cells were harvested by centrifugation at 5000xg for 10 min at 4 °C. The cell pellets were resuspended in TBS (50 mM TRIS pH 7.4 at room temperature, and 150 mM NaCl) and lysed by several passages through a M-110P microfluidizer (Microfluidics) operating at 20,000 PSI. The cell debris and insoluble material were pelleted by centrifugation at 20,000xg for 25 min at 4 °C. The membranes were pelleted by centrifugation at 100,000xg for 2 h at 4 °C. The pelleted membranes were resuspended in resuspension buffer (50 mM TRIS pH 7.4 at room temperature, 100 mM NaCl, 20 % Glycerol, and 5 mM B-mercaptoethanol) and homogenized prior to extracting membrane proteins by the addition of octyl glucoside (OG) at a final concentration of 5 %. After overnight extraction at 4 °C, insoluble material was pelleted by centrifugation (20,000xg for 20 min at 4 °C). The clarified supernatant was filtered using a 0.45-µm syringe filter (Whatman) and loaded onto a StrepTrap HP 5 mL column (Cytiva) pre-equilibrated in SPA (20 mM TRIS pH 7.4 at room temperature, 100 mM NaCl, 10 % Glycerol, and 0.025 % n-decyl-β-D-maltopyranoside (DM)) operating at a flow rate of 5 mL/min. The sample was backloaded with SPA containing 2 % OG buffer, washed with 5 column volume (CV) of the loading buffer, and eluted with the SPA containing 2.5 mM desthiobiotin. The eluent was concentrated using a 100 kDa MWCO Millipore concentrator (MilliporeSigma) and loaded onto a Superdex 200 Increase 10/300 GL column (GE Healthcare) equilibrated in GF buffer (20 mM TRIS pH 7.4 RT, 10 % glycerol, 100 mM NaCl, and 0.5 % C_8_E4). Peak fractions were pooled and concentrated. Prior to flash freezing in liquid nitrogen, glycerol was added to a final concentration of 20 %. Samples were stored at −80 °C.

*Anti-ALFA nB expression and purification.* The anti-ALFA nB expression plasmid was transformed into Shuffle T7 Express (NEB) that were previously transformed with the pRARE plasmid isolated from Rosetta cells (Agilent). Overnight culture and inoculation of TB was similar to the process described for AqpZ-ALFA, but the growth temperature was 30 °C. Once the OD reached between 0.6 and 0.8, protein expression was induced with the addition of 0.3 mM IPTG, and the temperature was lowered to 20 °C. The cells were harvested as described above for AqpZ-ALFA and resuspended in TBS. The resuspended cells were passed four times through a M-110P microfluidizer operating at 20,000 PSI. Imidazole was added to a final concentration of 20 mM before removing insoluble material by centrifugation at 40,000xg for 20 min at 4 °C. The clarified lysate was filtered before loading onto a His-Trap HP 5 mL column (Cytiva) pre-equilibrated in loading buffer (TBS containing 20 mM imidazole) at room temperature. The column was backloaded with the loading buffer, washed with 5CV of the loading buffer, and then eluted with a linear gradient to 100 % elution buffer (loading buffer containing 500 mM imidazole). Peak fractions were loaded onto a HiPrep 26/10 desalting column (GE Healthcare) pre-equilibrated in TBS. Fractions containing the desalted protein were pooled and digested with bdSENP1 (AddGene #104962, prepared following previously described protocols (Frey and Gorlich, 2014). After one hour of digestion at room temperature, the sample was loaded onto the HisTrap equilibrated in TBS, and the flowthrough containing tag-less nB was collected. Glycerol was added to a final concentration of 20 % prior to flash freezing in liquid nitrogen and storage at −80 °C.

*CryoEM of the AqpZ-ALFA-nB complex with CDL.* Samples for cryoEM were prepared by incubating purified AqpZ-ALFA with five-fold molar excess of nB in C_8_E_4_. After incubating for 30 min, the mixture was loaded onto a StrepTrap HP 5 mL column equilibrated in SPA. The complex was eluted with SPB and peak fractions were concentrated using a 100 kDa MWCO Millipore concentrator. The concentrated protein was loaded onto the Superdex 200 Increase 10/300 GL column (GE Healthcare) equilibrated in GF buffer. The AqpZ-ALFA-nB complex's peak fraction was pooled and concentrated to 10 mg/mL. 18:1 Cardiolipin (TOCDL, Avanti Polar Lipids) was added in a 5-fold molar excess ratio to the (tetrameric) AqpZ-ALFA-nB complex. Just before vitrification, the sample was filtered using a 0.2micron filter. The sample was applied to a Quantifoil R1.2/1.3, orthogonal array, 300 M Cu grids, blotted, and blotted at 5 °C and vitrified in liquid ethane using the Vitrobot Mark IV (Thermo Fischer Scientific).

CryoEM data was processed using RELION and CryoSparc. (Scheres, 2012, Punjani et al., 2017) A detailed data processing flowchart is shown in Fig. S3. 3081 movie stacks were first corrected by RELION implementing motion correction with bin2 and imported into CryoSPARC. Patch-based CTF estimation was used to determine CTF parameters for each micrograph. The blob picker was used to pick particles and identify templates after 2D classification. A particle set of 911,957 particles was picked using the template picker. After two rounds of 2D classification, 646,453 particles were selected for the downstream processing. Ab initio reconstruction was used to generate 3 initial volumes, which were used for heterogenous refinement. 388,221 particles were left after heterogeneous refinement and were subjected to homogenous refinement with application of D4 symmetry, which produced a map with a resolution of 2.0 Å (FSC 0.143). The particles and refined map were used for Bayesian polishing and 3D refinement in Relion. The polished particles were imported into CryoSPARC and used for homogeneous refinement with D4 symmetry. The polished particles were reconstructed to a map with a resolution of 1.9 Å (FSC 0.143). See Table S1 for details of image processing statistics.

*Model building, refinement, and validation of the single-particle cryo-EM structures.* The previously reported structures of AqpZ (PDB 1RC2, chain A) (Savage et al., 2003) and anti-ALFA nB bound to ALFA peptide (PDB 6I2G) (Gotzke et al., 2019) were docked into the cryo-EM map using Chimera. (Pettersen et al., 2004) The model was manually refined and CDL was modeled into the density using Coot. (Emsley and Cowtan, 2004) The final model was subjected to one round of real-space refinement using Phenix. (Adams et al., 2010) The final model refinement, statistics, and geometry can be found in Table S2. Figures were generated using ChimeraX (Pettersen et al., 2021) and Pymol (Schrödinger LLC., version 2.1).

## Code availability

Pymol scripts for grafting the ALFA tag onto AqpZ is available at https://github.com/LaganowskyLab.

## CRediT authorship contribution statement

**Lauren Stover:** Writing – original draft, Visualization, Methodology, Investigation, Formal analysis, Data curation, Conceptualization. **Hanieh Bahramimoghaddam:** Writing – original draft, Visualization, Methodology, Investigation, Formal analysis, Data curation, Conceptualization. **Lie Wang:** Writing – review & editing, Investigation, Formal analysis, Data curation. **Samantha Schrecke:** Writing – review & editing, Formal analysis, Conceptualization. **Gaya P. Yadav:** Writing – review & editing, Formal analysis, Data curation. **Ming Zhou:** Writing – review & editing, Visualization, Supervision, Funding acquisition, Formal analysis. **Arthur Laganowsky:** Writing – original draft, Visualization, Validation, Supervision, Project administration, Investigation, Funding acquisition, Formal analysis, Data curation, Conceptualization.

## Declaration of competing interest

The authors declare that they have no known competing financial interests or personal relationships that could have appeared to influence the work reported in this paper.

## Data Availability

AqpZ-ALFA-nB coordinates and maps have been deposited in the PDB and EMDB with accession code 8UY6 and EMD-42793, respectively. Other data associated with this manuscript is available upon reasonable request.
