# Supplementary material for: Grafting the ALFA tag for structural studies of aquaporin Z
Source: J Struct Biol X. 2024 Feb 2;9:100097. doi: 10.1016/j.yjsbx.2024.100097 (PMC10867769; doi:10.1016/j.yjsbx.2024.100097)
Supplement: Supplementary data 1 [file mmc1.pdf]

Supplementary Material

Grafting the ALFA tag for structural studies of Aquaporin Z

Lauren Stover,[1,†] Hanieh Bahramimoghaddam,[1,†] Lie Wang,[2] Samantha Schrecke,[2] Gaya P. Yadav,[3] Ming Zhou,[2] Arthur Laganowsky,[1,*]

[1] Department of Chemistry, Texas A&M University, College Station, TX 77843
[2] Verna and Marrs McLean Department of Biochemistry and Molecular Biology, Baylor College of Medicine, Houston, TX, 77030
[3] Laboratory for Biomolecular Structure and Dynamics (LBSD), Department of Biochemistry and Biophysics, Texas A&M University, College Station, TX 77843
† These authors contributed equally to this work
*Corresponding Author: ALaganowsky@chem.tamu.edu

Supplementary Figures

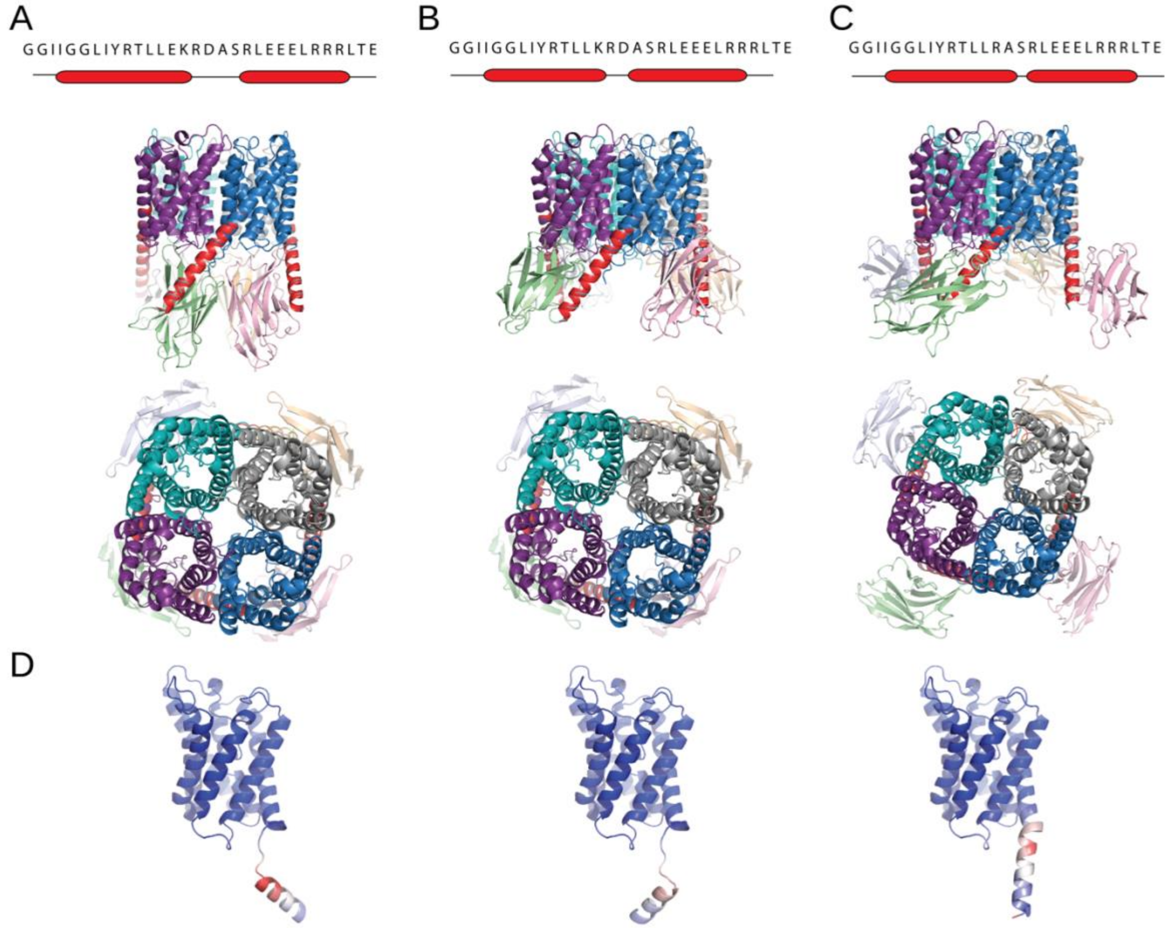

Figure S1. Theoretical grafts of the ALFA peptide onto the C-terminus of AqpZ. Shown are different fusions of the ALFA sequence onto the C-terminus of AqpZ. An alpha helical peptide (shown in red cartoon) with amino sequence shown was first aligned to the C-terminus of AqpZ. The ALFA tag in complex with nB (PDB 6I2G) was then aligned to get orientation of the nB. D) Shown are the AlphaFold[1, 2] models generated based on the fusion protein sequence and colored by confidence. Regions with the highest confidence are colored in blue.

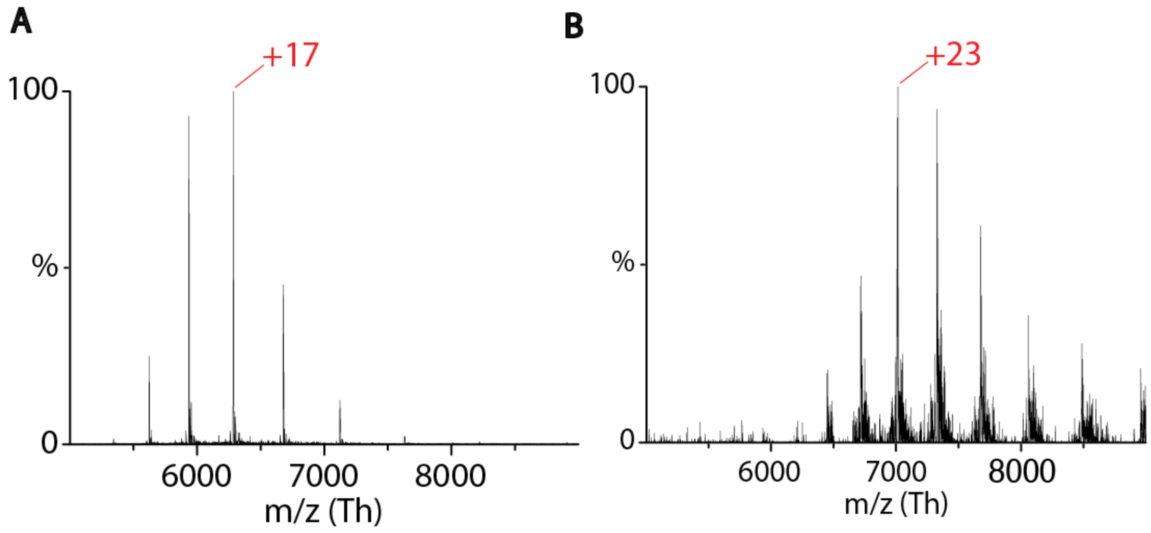

Figure S2. Mass spectra of AqpZ-ALFA and in complex with nB. (A-B) Native mass spectra of AqpZ-ALFA-St (panel A) and bound to nB (panel B), collected at room temperature.

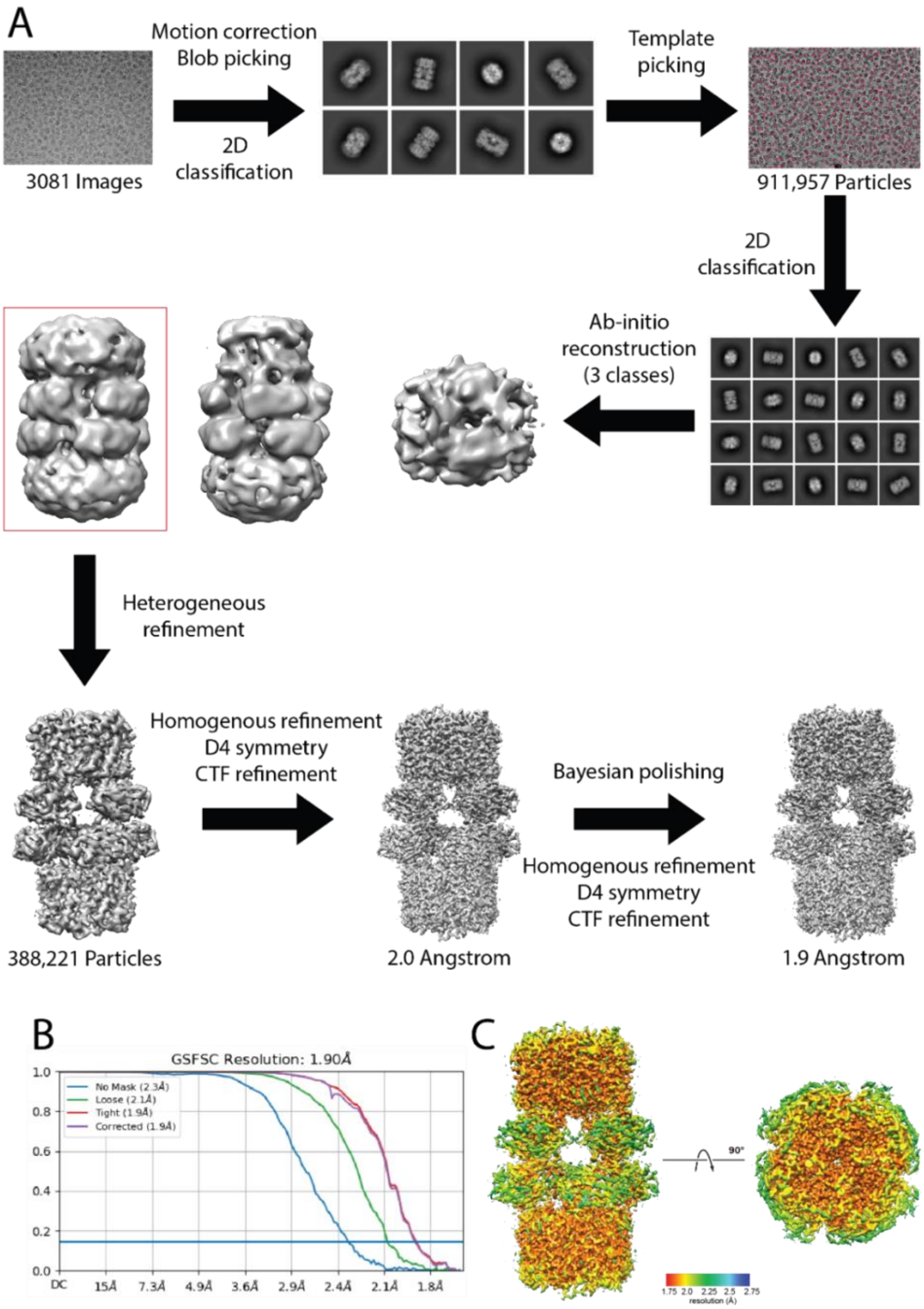

Figure S3. Single-particle cryo-EM analysis of AqpZ bound to nanobody and CL. (A) The workflow of data processing was carried out in cryoSPARC.[3, 4] A representative motion-corrected micrograph is shown along with a 50-nm scale bar. Particle selection was performed using the 2D templates generated by blob picker, followed by a 2D classification. Representative 2D class averages are shown, with the box edge corresponding to ~291 Å. Ab-initio reconstruction was performed followed by one round of heterogenous and then homogenous and CTF refinement with D4 symmetry imposed during refinement. (B) Fourier shell correlation curves for the final

map. The resolution of the reconstruction was determined by the FSC=0.143 criterion. (C) Local resolution map of the AqpZ complex shown in two orientations.

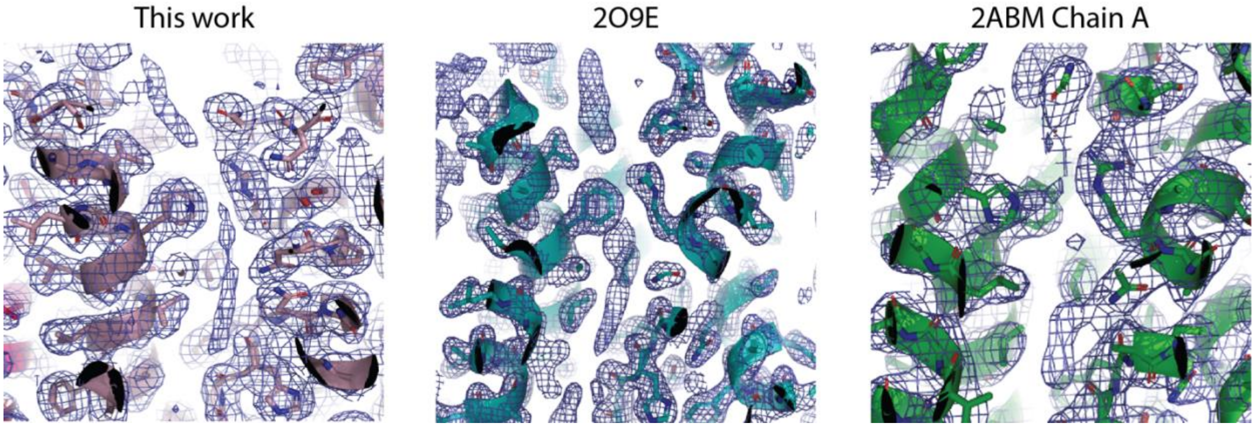

Figure S4. Comparison of density of the water channel within an AqpZ protomer. CryoEM density is contoured as described in figure 3. Electron density for the two other structures is contoured at 1 sigma. PDB codes are listed at the top.

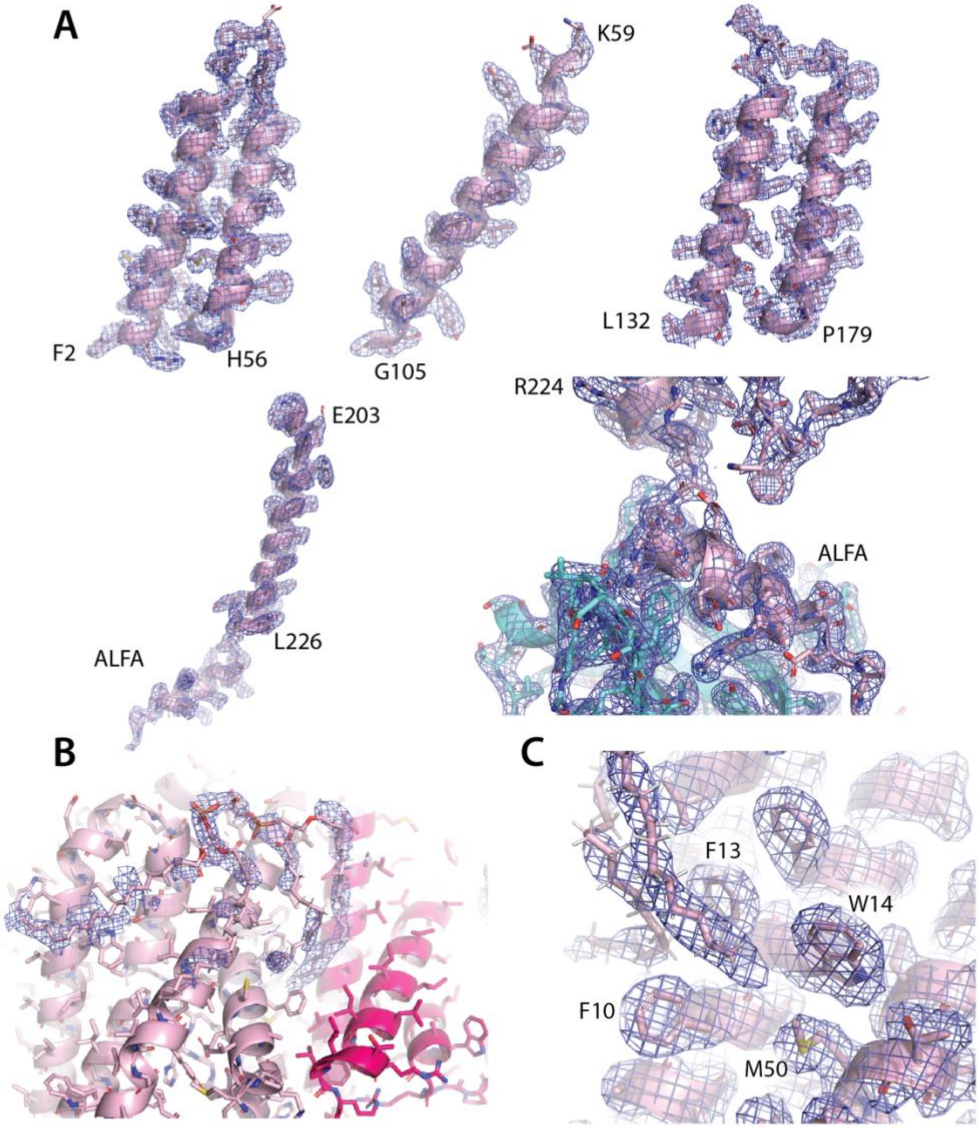

Figure S5. Cryo-EM density of the AqpZ complex. (A) Density (contoured at 6 sigma) and atomic model for various transmembrane helices (TMs) including the ALFA tag bound to the anti-ALFA nanobody (colored in cyan). (B-C) Density (contoured at 4 sigma) and atomic model for CDL. Shown in panel C is a view of the acyl chain that enters a grove at the subunit interface. Figure was prepared using Pymol.[5]

Supplementary Tables

Table S1. Statistics of cryo-EM data collection and processing.

	AqpZ-nB complex
Microscope	Krios G4 (Texas A&M University)
Magnification	105,000
Voltage (kV)	300
Spherical aberration (mm)	2.7
Detector	K3 (Gatan)
Camera mode	Super Resolution Counting
Exposure rate (e^-/pixel/s)	14.638
Exposure Time	2.4 seconds
Total exposure (e^-/Å^2)	50.75
Defocus range (µm)	-1.0 to -2.5
Pixel size (Å)	0.416
Mode of data collection	AFIS (Aberration Free Image Shift)
Energy filter (Gatan BioContinuum)	15 eV (slit width)
Software for data collection	EPU
Number of micrographs	3,081
Symmetry imposed	D4
Box size (pixel)	350
Initial particle images (no.)	1,785,869
Final particle images (no.)	388,221
Map resolution, unmasked (Å)	2.3
Map resolution, masked (Å)	1.9
B-factor used for sharpening (Å^2)	89.2
EMD accession code	EMD-42793

Table S2. Statistics of cryo-EM model refinement and geometry.

Model	AqpZ-nB complex
Composition (#)	
Chains	24
Atoms	23864 (Hydrogens: 1248)
Residues	Protein: 2928 Nucleotide: 0
Water	0
Ligands	CL: 8
Bonds (RMSD)	
Length (Å) (# > 4σ)	0.003 (0)
Angles (°) (# > 4σ)	0.533 (0)
MolProbity score	1.72
Clash score	8.37
EMRinger Score	5.48
Ramachandran plot (%)	
Outliers	0.28
Allowed	3.59
Favored	96.13
Rama-Z (Ramachandran plot Z-score, RMSD)	
whole (N = 2896)	0.86 (0.16)
helix (N = 1432)	1.02 (0.14)
sheet (N = 480)	0.67 (0.24)
loop (N = 984)	0.04 (0.22)
Rotamer outliers (%)	1.00
Cβ outliers (%)	0.00
Peptide plane (%)	
Cis proline/general	0.0/0.0
Twisted proline/general	0.0/0.0
CaBLAM outliers (%)	1.96

ADP (B-factors)		
Iso/Aniso (#)	22616/0	
min/max/mean		
Protein	29.18/134.00/65.93	
Ligand	88.12/141.90/118.42	
Occupancy		
Mean	1.00	
occ = 1 (%)	100.00	
Data		
Box		
Lengths (Å)	104.00, 104.00, 157.25	
Angles (°)	90.00, 90.00, 90.00	
Supplied Resolution (Å)	1.9	
Resolution Estimates (Å)	Masked	Unmasked
d FSC (half maps; 0.143)	2.0	2.0
d 99 (full/half1/half2)	2.6/2.4/2.4	2.6/2.2/2.2
d model	2.4	2.3
d FSC model (0/0.143/0.5)	1.9/1.9/2.0	1.9/1.9/2.1
Map min/max/mean	-0.28/0.87/0.02	
Model vs. Data		
CC (mask)	0.93	
CC (box)	0.83	
CC (peaks)	0.80	
CC (volume)	0.92	
Mean CC for ligands	0.53	

Supplementary References

1. Jumper, J., Evans, R., Pritzel, A., Green, T., Figurnov, M., Ronneberger, O., Tunyasuvunakool, K., Bates, R., Zidek, A., Potapenko, A., Bridgland, A., Meyer, C., Kohl, S. A. A., Ballard, A. J., Cowie, A., Romera-Paredes, B., Nikolov, S., Jain, R., Adler, J., Back, T., Petersen, S., Reiman, D., Clancy, E., Zielinski, M., Steinegger, M., Pacholska, M., Berghammer, T., Bodenstein, S., Silver, D., Vinyals, O., Senior, A. W., Kavukcuoglu, K., Kohli, P. & Hassabis, D. (2021). Highly accurate protein structure prediction with AlphaFold. Nature. **596**, 583-589. https://doi.org/10.1038/s41586-021-03819-2.
2. Senior, A. W., Evans, R., Jumper, J., Kirkpatrick, J., Sifre, L., Green, T., Qin, C., Zidek, A., Nelson, A. W. R., Bridgland, A., Penedones, H., Petersen, S., Simonyan, K., Crossan, S., Kohli, P., Jones, D. T., Silver, D., Kavukcuoglu, K. & Hassabis, D. (2020). Improved protein structure prediction using potentials from deep learning. Nature. **577**, 706-710. https://doi.org/10.1038/s41586-019-1923-7.
3. Punjani, A., Rubinstein, J. L., Fleet, D. J. & Brubaker, M. A. (2017). cryoSPARC: algorithms for rapid unsupervised cryo-EM structure determination. Nat Methods. **14**, 290-296. https://doi.org/10.1038/nmeth.4169.
4. Scheres, S. H. W. (2012). RELION: Implementation of a Bayesian approach to cryo-EM structure determination. Journal of Structural Biology. **180**, 519-530. https://doi.org/https://doi.org/10.1016/j.jsb.2012.09.006.
5. Schrödinger, L. a. W. D. (2020-05-20). PyMol v2.4.0. http://www.pymol.org/pymol
